# The Short Form Score for the Assessment and Quantification of Chronic Rheumatic Affections of the Hands in Daily Clinical Routines—Its Sensitivity to Change and Preliminary Patient Relevant Variation Values: A Pilot Study

**DOI:** 10.3389/fmed.2017.00006

**Published:** 2017-01-27

**Authors:** Ulrike Stummer, Bernhard Rintelen, Burkhard F. Leeb

**Affiliations:** ^1^Second Department of Medicine, Center for Rheumatology, State Hospital Stockerau, Stockerau, Austria; ^2^Karl Landsteiner Institute for Clinical Rheumatology, Stockerau, Austria; ^3^Department for Rheumatology and Immunology, Medical University of Graz, Graz, Austria

**Keywords:** SF-SACRAH, hand impairment, assessment methods, rheumatoid arthritis, hand osteoarthritis

## Abstract

**Objective:**

The SF-SACRAH was developed to assess the involvement of the hand in rheumatoid arthritis (RA) and hand osteoarthritis (HOA) patients in daily clinical routines. In this pilot study, its sensitivity to change will be assessed longitudinally, and preliminary thresholds for patient relevant changes are derived.

**Methods:**

Ninety-nine outpatients suffering from HOA (*n* = 55) or RA (*n* = 44) completed the SF-SACRAH once initially. After approximately 3 months, patients repeated the SF-SACRAH. At both visits, patients rated their satisfaction (PATSAT) with the state of their disease (1 = very good to 5 = unsatisfactory). For assessing its sensitivity to change, SF-SACRAH changes in patients with stable, improving, or worsening conditions according to PATSAT were calculated in HOA and RA patients. The respective medians and highest values were used to estimate patient relevant variation values. SF-SACRAH changes and positive or negative PATSAT changes in HOA as well as RA patients were analyzed by applying the Kruskal–Wallis test. In RA patients, the DAS28 was also calculated. Spearman’s rho was calculated to correlate SF-SACRAH changes with the EULAR response criteria.

**Results:**

In HOA and RA patients, a statistically high correlation between PATSAT changes and SF-SACRAH values was revealed (*p* < 0.0001 in HOA and *p* < 0.01 in RA patients, respectively). The median changes in SF-SACRAH in patients with improving, stable, or worsening conditions according to PATSAT were HOA patients: PATSAT improving: ΔSF-SACRAH −1.6, PATSAT stable: ΔSF-SACRAH +0.8, PATSAT worsening: ΔSF-SACRAH +1.0; RA patients: PATSAT improving: ΔSF-SACRAH −0.9, PATSAT stable: ΔSF-SACRAH +0.2, PATSAT worsening: ΔSF-SACRAH +0.8. In RA patients, there is a moderate, but significant, correlation between DAS28 EULAR response criteria and SF-SACRAH changes (ΔDAS28 improving >0.6: ΔSF-SACRAH −0.4, ΔDAS28 <0.6: ΔSF-SACRAH +0.0, ΔDAS28 worsening >0.6: ΔSF-SACRAH +0.5; *r* = 0.433, *p* < 0.01).

**Conclusion:**

The SF-SACRAH constitutes a reliable tool for the assessment of hand impairment in patients with chronic rheumatic diseases. It proved to be sensitive to change in this short-term evaluation in both HOA and RA patients. Additionally, preliminary patient variation values for improvement (−1.60) and deterioration (+1.0) could be derived.

## Introduction

Hand osteoarthritis (HOA) and rheumatoid arthritis (RA) are the disorders seen in routine rheumatology practice that most frequently lead to impaired hand function. This symptom is arguably one of the most decisive determinants for a patient’s well-being and ability to work (1, 2). We developed a Score for the Assessment and Quantification of Chronic Rheumatic Affections of the Hands (SACRAH) (3), which, alongside the Australian/Canadian osteoarthritis hand index (4), appeared to show the lowest diversity ratio and the highest percentage of linked internal classification of functioning, disability, and health in the available questionnaires for HOA (5). However, none of these questionnaires meet the requirements for use in daily clinical routines, as they are often comprised of too many questions, are too difficult or time consuming to calculate, or both. Moreover, it was shown that patients’ perspectives were related to the number of joints with a limited range of motion, but with no other symptoms. As such, patients’ opinions regarding joint function should be given a more prominent position in disease management (6). The M-SACRAH, developed from the SACRAH by reducing the number of questions, appeared to be sensitive to change after surgical intervention in rheumatoid hands (4, 7). Therefore, we pursued the objective of further developing a practical tool for the assessment of the involvement of the hand in rheumatic disorders. These efforts resulted in the Short Form Score for the Assessment and quantification of Chronic Rheumatic Affections of the Hands (SF-SACRAH), which is based on patients’ self-reports concerning function, stiffness, and pain (8). The SF-SACRAH comprises five questions: three questions on function pertaining to “locking/unlocking a door,” “fastening/unfastening a zip,” and “turning the pages of a newspaper”; one question on stiffness asking “how was your joint stiffness immediately after waking up in the morning”; and finally one question on pain asking about “pain in the hands at rest in the evening.” For the three questions concerning function, a score of 0 means “possible without any difficulty,” while a score of 10 means “impossible”; concerning stiffness a value of 0 indicates “no stiffness” and 10 “unbearable stiffness” or similar; in the last question concerning pain, a score of 0 indicates “no pain” and 10 “unbearable pain.” The SF-SACRAH appeared to provide high internal consistency (standardized item alpha 0.731 and 0.837, respectively) (8). Its Likert scales format from 0 to 10 ensures easy completion and quick calculation in less than 1 min. This also makes it easy to use for non-rheumatologists.

It is the aim of this short-term pilot study to prove SF-SACRAH’s sensitivity to change under routine conditions, as it had already been shown for both other SACRAH questionnaires (3, 9). This is designed to enable longitudinal observations of functional hand impairment in daily routines and potential observations by electronic monitoring instruments (10, 11). Moreover, preliminary thresholds for patient relevant improvement and deterioration should be derived.

## Patients and Methods

For this pilot study, HOA patients diagnosed according to the 2009—EULAR recommendations using the patient’s history, clinical aspects, hand X-rays, and normal acute phase reactions (12), as well as RA patients diagnosed according to the 1987—ARA criteria for RA (13) were asked to complete the SF-SACRAH questionnaire at two consecutive appointments with the outpatient clinic. Patients were not included if they had any other disorder or were taking any medication that potentially influenced hand function, for example, fibromyalgia, neurological disorders, peripheral vascular diseases, or the intake of aromatase inhibitors. Between the two visits, HOA patients were treated with a SYSADOA and analgesics or NSAIDs on demand, RA patients with conventional or biological DMARDs, some with glucocorticoids and all with NSAIDs on demand.

To obtain another patient-related parameter, all patients were asked to rate their current satisfaction with the state of their disease (PATSAT) according to the Austrian school marking system (1 = excellent, 2 = good, 3 = average, 4 = adequate and 5 = unsatisfactory or, corresponding to the Anglo-American school system: 1 = excellent, 2 = good or above average, 3 = average, 4 = below average or poor and 5 = unsatisfactory or fail). The Austrian school marking system resembles a Likert scale and is well established, which makes misinterpretations by the patients highly unlikely. Since no consistent tool has been proven to measure patients’ satisfaction with their state of disease in HOA or in RA patients, PATSAT was chosen to in order to derive a patient dependent parameter for the subjective disease status. A patient’s global assessment of the medical condition on a 100-mm visual analog scale [VAS of Patient’s Global Assessment (VASPGA)] was not conducted as an alternative since it already forms part of the DAS28. Moreover, PATSAT was shown to have a significant correlation with composite disease activity indices, as well as with a patient’s attitude toward an increase or reduction in RA therapy (14).

We defined improvement in PATSAT as an observable positive change of least 1 point in PATSAT between visit 1 and visit 2, stability as no change in PATSAT, and deterioration as a negative change in PATSAT of at least 1 point between the two visits.

In RA patients, the Disease Activity Score, including a 28 joint count and the erythrocyte sedimentation rate (ESR) first hour (DAS28ESR), was calculated (15) to obtain insights into disease activity and to correlate the functional status of the hands measured by SF-SACRAH and to obtain insights to the change of SF-SACRAH according to RA disease activity defined to be as stable or improving according to the EULAR response criteria (16). For the calculation of DAS28ESR, the 28 predefined joints were assessed with regard to tenderness and swelling by experienced professionals. In addition, VASPGA and the ESR (first hour) were recorded to calculate DAS28ESR at both visits.

Changes in SF-SACRAH and PATSAT between visit 1 and visit 2 were calculated for each HOA and RA individual patient and in addition, the DAS28 change for each RA patient. Subsequently, the relationship between SF-SACRAH changes and improvement, stability or deterioration of PATSAT was determined in all groups by applying the Kruskal–Wallis test, as normal distribution could not be ascertained previously. Spearman’s rho was applied to correlate changes in SF-SACRAH with DAS28ESR EULAR response criteria in RA patients. Median changes in SF-SACRAH in relation to PATSAT improvement, stability, or deterioration were additionally calculated in order to estimate patient relevant SF-SACRAH changes. The highest median difference in SF-SACRAH for improvement or deterioration according to PATSAT will be used to define the preliminary patient relevant variation value. Since this study is considered a pilot study, no sample size calculation was performed.

As this study constitutes an analysis of existing, routinely compiled data and no direct personal patient data were processed by the authors; Austrian law does not provide a basis for engagement of an ethics committee. Nevertheless, the study was informally brought to attention of the Lower Austrian Ethics Committee, which did not express any concerns related to this patient evaluation.

## Results

### All Patients

Fifty-five patients suffering from HOA and 44 patients suffering from RA completed the SF-SACRAH questionnaire during two consecutive appointments at the outpatient clinic. For patient’s characteristics, see Table 1. The median time period between the two visits was 3.0 (min–max: 0.5–8) months in the HOA group and 3.5 (min–max: 0.5–8.0) months in the RA group. The SF-SACRAH for HOA patients at visit 1 was a median of 2.2 and then 2.0 during visit 2, whereas for RA patients, the median at visit 1 appeared to be 2.4 and then 2.6 during visit 2 (see Table 1).

**Table 1:** **Patient’s characteristics**.

	RA (*n* = 44)		HOA (*n* = 55)	
Age median (min–max)	61.0 (32–72)		61.5 (46–73)	
Gender (female %)	90.9		87.3	
RF+ (%)	59.1			
Disease duration months median (min–max)	59 (4–215)		24 (4–336)	
Δ visit 1 and 2 months median (min–max)	3.5 (0.5–8.0)		3.0 (0.5–8.0)	

	**Visit 1**	**Visit 2**	**Visit 1**	**Visit 2**

DAS28 median (min–max)	3.96 (1.40–6.96)	3.93 (0.77–6.50)	–	–
PATSAT (1–5) median (min–max)	3 (1–5)	3 (1–5)	3 (1–5)	3 (1–5)
SF-SACRAH median (min–max)	2.4 (0.0–7.8)	2.6 (0.0–7.6)	2.2 (0.0–9.0)	2.0 (0.0–8.0)
SJC (out of 28) median (min–max)	2 (0–15)	2 (0–16)	0 (0–8)	0 (0–3)
TJC (out of 28) median (min–max)	4 (0–28)	3 (0–26)	1 (0–19)	1 (0–19)
VASPGA mm (0–100) median (min–max)	50 (0–98)	38 (0–100)	30 (0–78)	31 (0–74)
ESR first hour median (min–max)	18 (1–88)	21 (1–66)	11 (2–74)	12 (3–74)

*DAS28, Disease Activity Score including 28 joints; ESR, erythrocyte sedimentation rate; HOA, hand osteoarthritis; max, maximum; min, minimum; mm, millimeter; PATSAT, patient’s satisfaction with the disease activity state; RA, rheumatoid arthritis; RF, rheumatoid factor; SJC, swollen joint count out of 28; SF-SACRAH, Short Form of the Score of Assessment of Chronic Rheumatic Affections of the Hand; TJC, Tender Joint Count out of 28; VAS, visual analog scale; VASPGA, VAS of patient’s global assessment*.

### SF-SACRAH in HOA Patients

**Figure 1:**
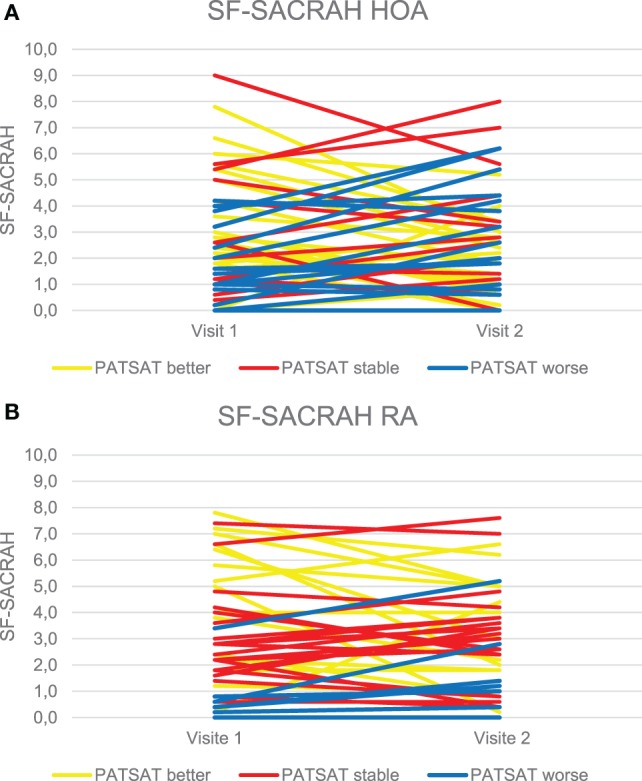
**(A)** Changes in the SF-SACRAH between visit 1 and visit 2 in hand osteoarthritis (HOA) patients. **(B)** Changes in the SF-SACRAH between visit 1 and visit 2 in rheumatoid arthritis (RA) patients.

**Figure 2:**
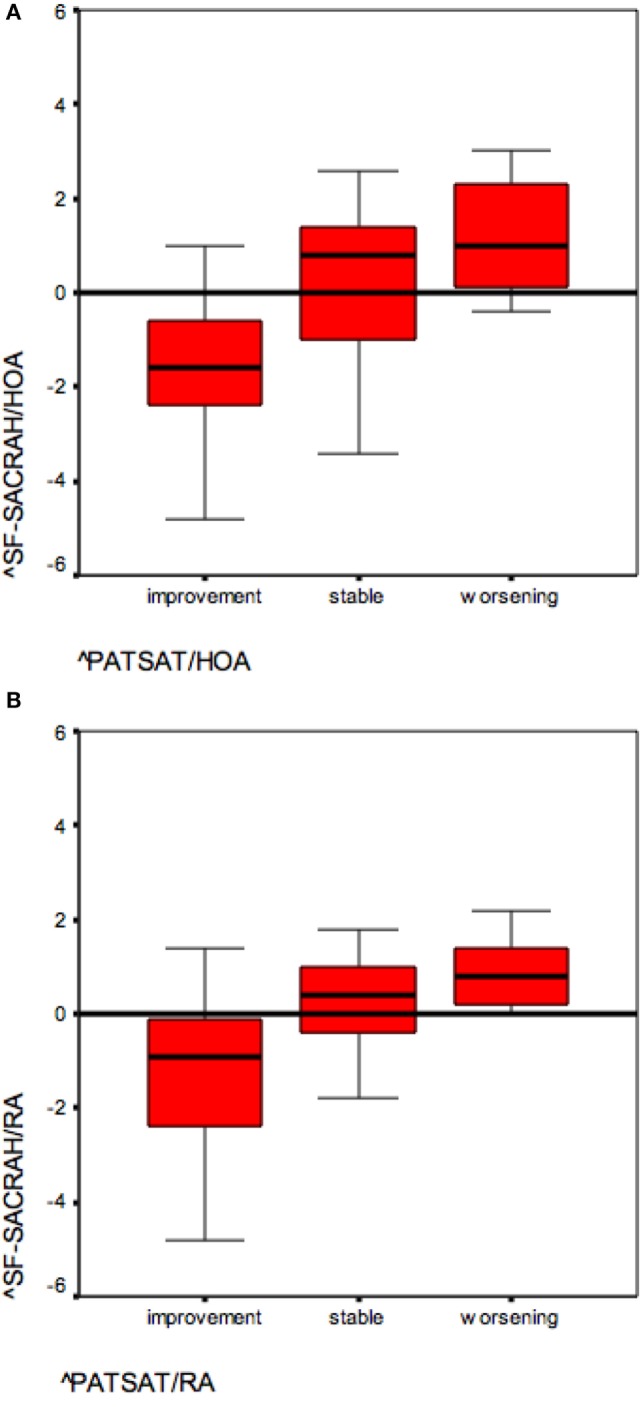
**(A)** SF-SACRAH changes in improving and worsening hand osteoarthritis (HOA) patients (reference PATSAT). **(B)** SF-SACRAH changes in improving and worsening rheumatoid arthritis (RA) patients (reference PATSAT).

The SF-SACRAH improved in 57% of HOA patients, was stable in 2%, and worse in 41% of patients (Figure 1A). In HOA patients, the median PATSAT amounted to 3.0 at visit 1 and also at visit 2. In the PATSAT improvers, the median SF-SACRAH change was −1.6, in PATSAT-stable patients +0.8, while in the patients with worsening PATSAT, the respective SF-SACRAH change was +1.0 (Table 2; Figure 2A). When applying the Kruskal–Wallis test, changes in PATSAT and SF-SACRAH were found to be of statistically high significance (*p* < 0.0001).

**Table 2:** **SF-SACRAH changes according to PATSAT changes in HOA patients**.

HOA (*n* = 55)	PATSAT improving	PATSAT stable	PATSAT worsening
% patients	48	24	28
Δ SF-SACRAH median (min; max)	−1.6 (−4.8; +1.8)	+0.8 (−3.4; +2.6)	+1.0 (−0.4; +3.0)

*HOA, hand osteoarthritis; n, number of patients; max, maximum; min, minimum; PATSAT, patient’s satisfaction with the disease activity state; SF-SACRAH, Short Form of the Score of Assessment of Chronic Rheumatic Affections of the Hand*.

### SF-SACRAH in RA Patients

The SF-SACRAH improved in 48%, was stable in 9%, and worse in 43% of RA patients (Figure 1B). As in the HOA patients, the RA patients demonstrated a median PATSAT of 3.0 at visit 1 and also at visit 2. The median SF-SACRAH change between the two visits was −0.9 in the patients showing improvement according to PATSAT, +0.2 in stable patients, and +0.8 in the worsening group (Table 3; Figure 2B). When applying the Kruskal–Wallis test, the RA patient group also revealed a statistically significant relationship between the SF-SACRAH and PATSAT changes (*p* < 0.01).

**Table 3:** **SF-SACRAH changes according to PATSAT changes in RA patients**.

RA (*n* = 44)	PATSAT improving	PATSAT stable	PATSAT worsening
% patients	36	48	16
Δ SF-SACRAH median (min; max)	−0.9 (−4.8; +1.4)	+0.2 (−1.8; +1.8)	+0.8 (0.0; +2.2)

*n, number of patients; max, maximum; min, minimum; PATSAT, patient’s satisfaction with the disease activity state; RA, rheumatoid arthritis; SF-SACRAH Short Form of the Score of Assessment of Chronic Rheumatic Affections of the Hand*.

At visit 1, the median DAS28 in the RA patients amounted to 3.96, indicating moderate disease activity. There was no change in median disease activity at visit 2 (see Table 1).

Patients achieving at least a moderate EULAR response (DAS28 positive change ≥0.6) showed a median SF-SACRAH reduction of −0.4 (min–max: −4.8; +0.8), stable patients demonstrated constant SF-SACRAH of ±0.0 (−1.8; +1.4), and when applying the reverse EULAR response criteria for RA worsening (patients with DAS28 negative changes ≥0.6), the respective SF-SACRAH change amounted to +0.5 (−3.4; +4.2). The correlation between the DAS28 changes and SF-SACRAH changes was found to be moderate but statistically significant, according to Spearman’s rank correlation coefficient (*r* = 0.433; *p* < 0.01).

### Preliminary Patient Relevant Variation Values for SF-SACRAH

As shown in Tables 2 and 3, according to SF-SACRAH, we propose the following preliminary patient relevant variation values: for improvement a positive change of −1.6 and for deterioration a negative change of at least +1.0.

## Discussion

During this pilot study, the SF-SACRAH proved to be an appropriate tool for assessing the severity of hand impairment due to HOA and RA and for according this impairment a numerical value. Additionally, the questionnaire’s sensitivity to change in relation to patient-related outcomes—in this case PATSAT—as well as composite indexes—in this study the DAS28ESR—could be demonstrated. These findings support the use of SF-SACRAH in clinical routine and investigative rheumatology to obtain greater understanding of affections of the hands in chronic rheumatic diseases such as HOA and RA. It is easily applicable as a patient-related outcome measure and can be completed by the patient in less than 1 min. The value is extremely easy to calculate by adding five whole numbers and subsequently dividing the total by 5.

Regarding improvement, stability, and deterioration, the patient relevant changes revealed different results for HOA and RA patients. This finding may be due to the relatively small patient numbers, but could be also be disease related, which should inspire further research.

Interestingly, the values indicating patient relevant changes were numerically higher in HOA patients. Moreover, the numerical change in cases of deterioration is smaller than in cases of improvement. Another finding in RA patients seems worth mentioning: DAS28 changes which would meet the threshold for a moderate EULAR response (16) do not comply with the preliminary patient relevant SF-SACRAH changes. This finding corresponds to previous results, which showed a significant correlation between DAS28 and SF-SACRAH in moderate and high disease activity, but an insignificant one in remission and low disease activity, a state which most of the patients included in this study had reached (17). Another RA study emphasized the lack of agreement between the patient’s and physician’s perception of RA activity changes. It also focused on the differences between improvement and deterioration in patient-related disease activity assessments (18).

The derived preliminary thresholds for patient relevant changes in hand impairment in RA and HOA certainly require further evaluation but may serve as a guideline for the future application of the score in routine rheumatology and also for research purposes. Patient-related outcomes will become increasingly important as it is likely that in future, rheumatic disease management will be facilitated by greater use of innovative electronic tools (19). To enable this, short and reliable questionnaires and thresholds for relevant disease activity changes form fundamental prerequisites. The SF-SACRAH proved to serve as an appropriate and simple tool (including for non-rheumatologists) to assess both the extent of the involvement of the hand and also hand impairment in HOA and RA. It will also serve as a useful tool to monitor the condition over time.

This study has several limitations. The first and most important is the small number of patients included. Nonetheless, sensitivity to change could be demonstrated. The preliminary thresholds for improvement or deterioration in hand function according to SF-SACRAH should therefore be used with caution and further studies are mandatory. Second, PATSAT is not part of the Outcome Measures in Rheumatology (OMERACT) recommended instruments for HOA or RA assessment. But as stated earlier, we searched for an additional patient-related assessment for HOA and RA patients. The OMERACT instrument VASPGA is used in the DAS28ESR as mentioned in the methods. PATSAT was shown to be significantly correlated with composite disease activity indices as well as to a patient’s attitude toward increase or reduction of therapy (14). Research into patient-related outcomes such as the SF-SACRAH requires innovative approaches. Relating a functional score to a clinical score for disease activity may not be the most appropriate way to demonstrate sensitivity to change or to search for improvement or deterioration, as they should constitute different parts of one assessment. Third, this is a short-term investigation, and therefore, we cannot reach any conclusions on whether our results are consistent in the long term or even for an electronic survey. Fourth, SF-SACRAH and its mother instruments are not examined in different hand disability stages. However, we are convinced that each patient with compromised function of his or her hands has to reach a better functional stage through our treatment. The SF-SACRAH could be an appropriate tool to monitor these efforts, but more research is mandatory.

Neither the application of any questionnaire nor any index can substitute for careful clinical patient examination (20). However, since rheumatologists focus increasingly on individualized treatment regimens, questionnaires may provide useful ways to identify patients requiring particular attention. No single measure can serve as a “gold standard” in the treatment of individual patients in most rheumatic diseases (21). The SF-SACRAH constitutes one easy option for routine monitoring, enabling health-care professionals to obtain reliable information about the disease course and focusing on hand impairment.

## Conclusion

- SF-SACRAH proved its reliability and sensitivity to change.
- Preliminary patient relevant thresholds could be derived for RA and HOA patients, namely, −1.6 for improvement and +1.0 for deterioration.

## Author Contributions

US designed the study, participated in data acquisition, and wrote the manuscript; BR designed the study, participated in data acquisition, and manuscript preparation; BL designed the study and participated in the statistical analyses and manuscript finalization.

## Conflict of Interest Statement

The authors declare that the research was conducted in the absence of any commercial or financial relationships that could be construed as a potential conflict of interest.
